# Efficacy and safety of traditional Chinese medicine and Western medicine in Alzheimer’s disease: a systematic review and meta-analysis

**DOI:** 10.3389/fneur.2025.1607945

**Published:** 2025-09-15

**Authors:** Qi Wang, De-Long Wang, Xiu-Chu Zhang, Xin-Yu Jiang, Huan-Ning Jiang, Xue-Ying Yang, Te Zhang, Yu-Ying Lv, Quan Li

**Affiliations:** ^1^College of Basic Medicine, Heilongjiang University of Chinese Medicine, Harbin, China; ^2^Key Laboratory of Neurobiology (Encephalopathy) of Clinical Acupuncture, The Second Affiliated Hospital of Heilongjiang University of Chinese Medicine, Harbin, China; ^3^Department of Acupuncture, The First Psychiatric Hospital of Harbin, Harbin, China; ^4^Department of Fourth Therapy, The First Psychiatric Hospital of Harbin, Harbin, China; ^5^Harbin Hospital of Traditional Chinese Medicine, Harbin, China

**Keywords:** Alzheimer’s disease, traditional Chinese medicine, meta-analysis, randomized controlled trial, treatment

## Abstract

**Objective:**

This study aimed to compare the efficacy and safety of traditional Chinese medicine (TCM) compounds with single Western medicines in treating Alzheimer’s disease (AD) through a systematic review and meta-analysis.

**Methods:**

In this study, we searched for randomized controlled trials on the treatment of AD with TCM compounds published before March 2025 in Chinese and English databases (PubMed, Embase, Cochrane, China National Knowledge Infrastructure, VIP, and Wanfang) and conducted a meta-analysis using Stata15.0 software.

**Results:**

A total of 23 studies were included, involving 2,035 participants (1,173 in the experimental group and 862 in the control group). Traditional Chinese herbal compounds showed good clinical efficacy and maintenance effects in the treatment of AD. The effective rate of TCM compounds in treating AD was higher than that of Western medicine (relative risk ratio = 1.19, 95% CI: 1.04–1.37, *p* = 0.009). In terms of the Alzheimer’s Disease Assessment Scale-Cognitive and Hierarchic Dementia Scale-Revised scores, TCM compounds were superior to Western medicine (standardized mean difference = −0.22, 95% CI: −0.40−−0.05). There were no significant differences between the two groups in the Mini-Mental State Examination or Activities of Daily Living scores. Additionally, there were no significant differences in adverse reactions between the TCM compounds and Western medicine groups.

**Conclusion:**

The present research indicates that TCM compounds could be a promising therapeutic option for AD, demonstrating encouraging results in terms of efficacy and safety, particularly regarding certain cognitive functions.

.

## 1 Introduction

Alzheimer’s disease (AD) is a neurodegenerative disease that occurs in older adults and is characterized by cognitive dysfunction and personality and behavioral disorders (1). According to epidemiological statistics, the number of patients with AD in the world is increasing year by year, and China has the largest ageing population. Older adults in China account for 13.26% of the total population, and there are more than 8 million patients with AD; this number is expected to exceed 20 million by 2050 (2). In addition, AD is harmful. Studies have pointed out that AD has now become the fourth largest killer affecting human health, following cardiovascular disease, cerebrovascular disease, and cancer (3). Therefore, it is becoming increasingly important to prevent and treat dementia (the most common form of AD) in an ageing society (4).

Currently, many theories exist regarding the aetiology and pathogenesis of AD, such as the *β*-amyloid (Aβ) (5, 6) and Tau protein hypotheses (7). Although these hypotheses are related to AD, their role in the whole progression of the disease is not clear. Presently, drugs like donepezil and memantine are used to improve cognitive function and control mental symptoms (8). However, these drugs are only suitable to improve the cognitive function of patients with mild to moderate forms of the condition and cannot prevent or reverse the progression of the disease (9). AD belongs to the category of mental illness in traditional Chinese medicine (TCM), where it is known as ‘dementia.’ The pathogenesis of AD is ‘lack of marrow sea and disuse of mind’. Deficiency is the pathological foundation of AD, phlegm is the key to its pathology, and blood stasis is the pathological product (3). Spleen and kidney deficiency, phlegm turbidity, blood stasis, and toxin are the characteristics of its pathogenesis. Kidney deficiency is the root of AD, and spleen deficiency is its key (10, 11). Therefore, the treatment is based on syndrome differentiation.

Basic clinical studies of TCM in the treatment of AD have achieved certain results (12, 13). Xingnao Powder (14), Huanglian Jiedu Decoction (15), Tiaoxin Decoction (16), Shenghuang Yizhi Decoction (17), and other TCM compound studies have shown good effects on AD. However, the sample size of single clinical trials is often small, the test methods are different, and the quality is uneven. There is limited evidence on the effect of TCM compounds in treating AD and whether it has definite advantages compared with Western medicine. Therefore, through a systematic review and meta-analysis, this study examines the published randomized controlled trials of TCM compounds and single Western medicine in treating AD and discusses their relative effectiveness. It offers a foundation for the clinical application of TCM compounds to provide a reference for the formulation of an AD treatment plan.

## 2 Materials and methods

### 2.1 Literature retrieval strategy

The PubMed, Embase, Cochrane, China National Knowledge Infrastructure, VIP, and Wanfang databases were used for a systematic search for studies on the treatment of AD with TCM compounds published before 7 March 2025. Chinese search terms included the following: ‘Chinese medicine compound’, ‘Chinese medicine’, ‘Chinese herbal medicine’, ‘Alzheimer’s disease’, and ‘dementia.’ English search terms included the following: ‘Chinese medicine’, ‘Herb’, ‘traditional Chinese medicine’, ‘herbal formula’, ‘Alzheimer’s Dementia’, ‘AD’, ‘Alzheimer’s Diseases’, and ‘Alzheimer’s Type Dementia.’

### 2.2 Inclusion and exclusion criteria

Inclusion criteria: (1) research type—published randomized controlled trials in both Chinese and English literature; (2) participants—patients diagnosed with AD, with a detailed diagnostic basis; (3) intervention measures—TCM compounds (the control group was Western medicine); (4) outcome indicators—effective rate, baseline Mini-Mental State Examination (MMSE) (18) score, Alzheimer’s Disease Assessment Scale-Cognitive (ADAS-cog) (19) score, Activities of Daily Living (ADL) (20) score, and the Hasegawa Dementia Scale (HDS) score (21).

Exclusion criteria: (1) single Chinese medicines; (2) the treatment measures of the control group included non-Western medicine; (3) repeated reports or inability to extract valid data; (4) case reports, reviews, or meeting summaries; (5) experiments involving animals or corpses.

### 2.3 Information extraction and quality evaluation

Two researchers independently screened the literature, extracted data in strict accordance with the inclusion and exclusion criteria, and discussed and resolved any differences. The extracted contents included the first author, publication year, sample size, intervention measures, outcome indicators, and diagnostic criteria.

The improved Jadad rating scale (22) was used to evaluate the literature. This scale evaluates the quality of studies based on the random allocation method, whether allocation concealment occurred, if the blinding method was properly implemented, and whether participant withdrawal and dropout were described adequately. The literature with a total score of 4–7 indicated high-quality research, 1–3 indicated low-quality research, and 0 indicated it was not included in the study. The quality evaluation process was performed independently by two researchers, and the final score of the article was determined through discussion in cases of disagreement.

### 2.4 Statistical analysis

A meta-analysis was performed on the research data using Stata15.0 statistical software. Count data were estimated by the relative risk ratio (RR) value and its 95% CI, and measurement data were expressed by the standardized mean difference (SMD) and its 95% CI. The inconsistency (*I*^2^) (23) statistic was used to evaluate the heterogeneity caused by non-threshold effects; when *I*^2^ ≥ 50%, the DerSimonian and Laird random effect models were used for meta-merger; when *I*^2^ < 50%, the fixed effect model was used. In addition, sensitivity analyses were performed to evaluate the stability of the summary results by excluding studies individually. Potential publication bias was assessed by the significant asymmetry of funnel plots and the Egger’s test.

## 3 Results

### 3.1 Literature screening results

**Figure 1:**
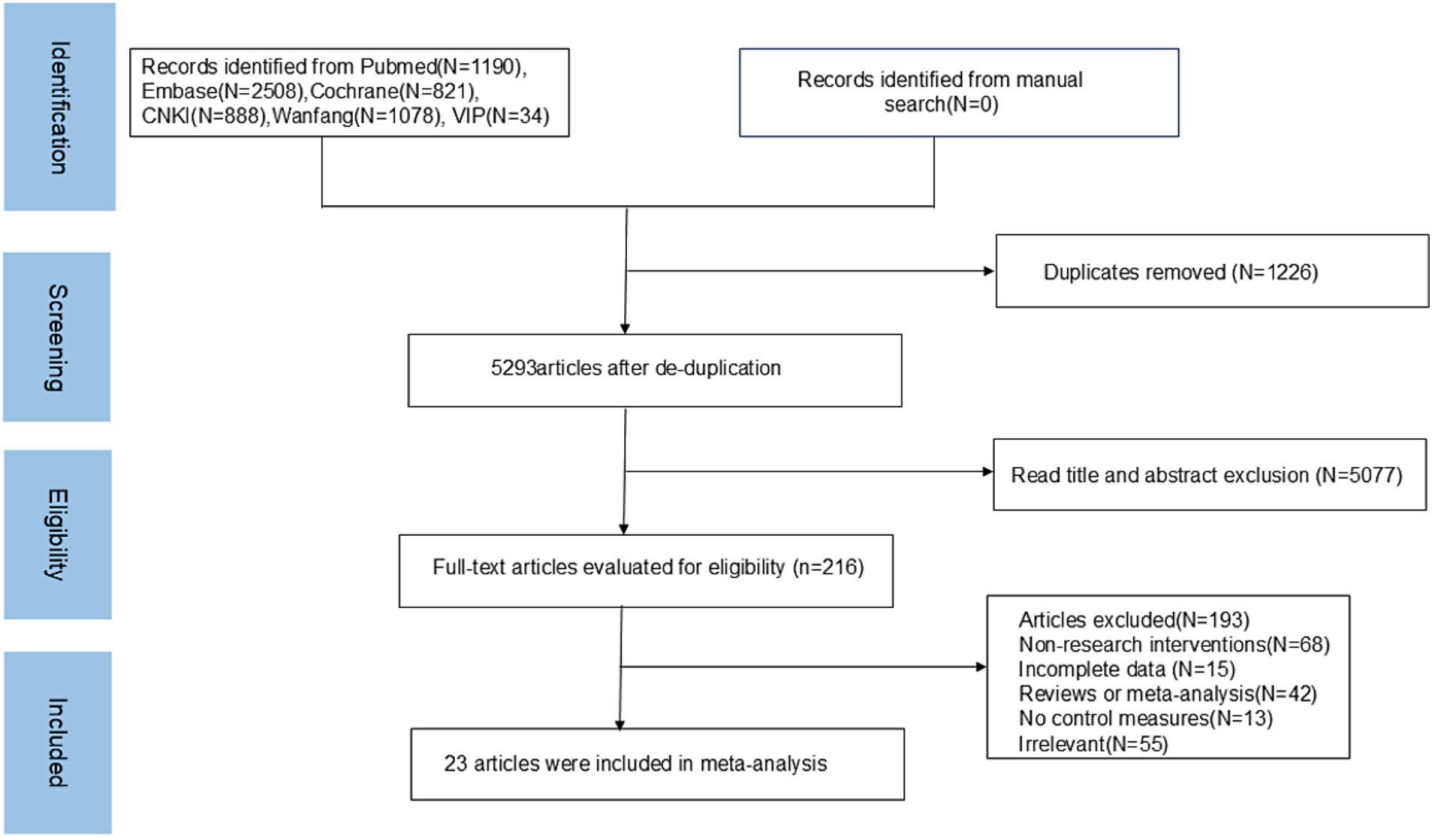
Literature screening process and results.

A total of 6,519 articles were retrieved in this study; 1,226 repetitive articles were excluded, 5,077 unrelated articles were excluded according to the title and abstract, and the remaining 216 articles were screened according to the full text. Finally, 23 articles were included in the meta-analysis. The literature screening process is shown in Figure 1.

### 3.2 Basic information of the included literature

The studies included a total of 1,735 participants (973 in the experimental group and 762 in the control group). Table 1 lists the basic characteristics of the included studies. The treatment measures of the experimental group involved a variety of TCM compounds, including Laozhifu Oral Liquid (24), Danggui Shaoyao Liquid (25, 26), Yizhi Jiannao Prescription (27–35), Shenghuang Yizhi (17, 36), Compound Heshouwu (37), Bushen Yiqi (38–40), Modified Shuyu Pill (41), Wenchi Prescription (42), Tiaobu Xinshen Prescription (43), Huanglian Jiedu Decoction (15), and Kangshuailing (44). The Western medicine treatment measures in the control group were primarily donepezil (15, 17, 26, 31, 32, 34, 35, 39–44), piracetam (29, 33, 38), Huperzine a (24, 28), Brain rehabilitation (27, 30, 37), nimodipine (25), and Pyritinol hydrochloride (36). The outcome indicators mostly involved efficiency (15, 17, 24–30, 33, 36–44), MMSE (17, 25–27, 29, 31–38, 40, 41, 43, 44), HDS (17, 24, 25, 27, 28, 36, 44), ADL (15, 17, 25, 29, 34–38, 41, 42, 44), and ADAS-cog (31, 34, 35, 40, 41).

**Table 1:** Basic characteristics of 14 studies included.

Author	Publishing time	Intervention measures		Sample size		Outcome index
		Experimental group	Control group	Experimental group	Control group	
Liu et al. (24)	2001	Laozhifu oral liquid	Huperzine a	20	15	Effective rate/HDS/Adverse reaction
Li et al. (25)	2002	Angelica peony liquid	Nimodipine	18	14	Effective rate/MMSE/ADL/HDS
Dong et al. (27)	2003	Qingnao Yizhiling	Brain rehabilitation	60	30	Effective rate/MMSE/HDS
Mu and Li (28)	2004	Naocong Decoction	Huperzine a	20	15	Effective rate/HDS
Meng et al. (29)	2005	Naohuandan	Piracetam	30	28	Effective rate/MMSE/ADL/Adverse reaction
Zhang et al. (30)	2006	Naokang instant granules	Brain rehabilitation	90	30	Effective rate
Zhang (36)	2006	Ingredient modified“rehmannia decoction”	Pyritinol hydrochloride	30	26	Effective rate/MMSE/ADL/HDS/Adverse reaction
Tian et al. (44)	2007	Kangshuailing	Donepezil	128	128	Effective rate/MMSE/ADL/HDS/Adverse reaction
Ding et al. (17)	2009	Shenghuang Yizhi Granules	Donepezil	33	30	Effective rate/MMSE/ADL/HDS
Chen et al. (37)	2010	Compound Heshouwu	Brain rehabilitation	120	29	Effective rate/MMSE/ADL
Liu and Dong (31)	2010	Puzzle brain health	Donepezil	10	10	MMSE/ADAS-cog
Zhu et al. (32)	2010	Puzzle brain health	Donepezil	20	20	MMSE
Shimeng et al. (26)	2011	Modified Danggui Shaoyao Powder	Donepezil	32	28	Effective rate/MMSE/Adverse reaction
Liu et al. (38)	2013	Tonifying kidney and resolving phlegm Yizhi	Piracetam	30	30	Effective rate/MMSE/ADL/Adverse reaction
Zhai et al. (33)	2013	Qi smart soup	Piracetam	30	30	Effective rate/MMSE
Zhang et al. (39)	2015	Yishen huazhuo soup	Donepezil	72	72	Effective rate/Adverse reaction
Wen et al. (40)	2016	Kidney-invigorating prescription	Donepezil	30	30	Effective rate/MMSE/ADAS-cog/Adverse reaction
Li (41)	2017	Modified Shuyu Pills	Donepezil	33	35	Effective rate/MMSE/ADAS-cog/ADL/Adverse reaction
Tan et al. (42)	2019	Wen Chi Fang	Donepezil	47	47	Effective rate/ADL/Adverse reaction
Lin et al. (43)	2020	Adjusting heart and kidney prescription	Donepezil	44	39	MMSE/Adverse reaction
Wang et al. (34)	2020	Jiannao Yizhi Recipe	Donepezil	30	30	MMSE/ADAS-cog/ADL/Adverse reaction
Yang (15)	2020	Coptidis decoction for detoxification	Donepezil	16	16	Effective rate/ADL/Adverse reaction
Chenggang (35)	2022	Modified Shenghui Decoction	Donepezil	30	30	MMSE/ADAS-cog/ADL/Adverse reaction

HDS, hasegawa dementia scale; MMSE, Mini Mental State Examination, mini-mental state examination; ADAS-cog, Alzheimer’s Disease Rating Scale-Cognitive, Alzheimer’s disease cognitive function rating scale; ADL, Activities for Daily Living subscale, daily life ability scale.

### 3.3 Quality evaluation of the included studies

The Jadad scale was used to evaluate the quality of the included literature, as shown in Table 2. The specific scores of the 23 articles included are as follows: a total of eight articles scored 4–7 points (comprising two research articles with a score of seven points, 1 article with a score of 5 points and five articles with a score of 4 points), representing high-quality literature; a total of 15 articles scored ≤3 comprising five articles with 3 points and 10 articles with 2 points (representing low-quality literature). Generation of random sequences: 10 studies used randomization and described the randomization method correctly. Randomization concealment: 19 studies only described the use of the random number method or random number table method and other random allocation schemes; however, they did not mention whether this method made it impossible for clinicians and participants to predict the allocation sequence. Three studies used opaque envelopes for randomization concealment, and one study was controlled by computer. The use of the blinding method indicated that two studies used a consistent placebo tablet. A total of 10 studies described the number of and reasons for withdrawal or loss of follow-up.

**Table 2:** Quality evaluation of included studies.

Author	Year	Random	Randomized hiding	Blind method	Withdrawn/lost	Total score
Liu et al. (24)	2001	1	1	0	0	2
Li et al. (25)	2002	1	1	0	0	2
Guiying et al. (27)	2003	1	1	0	0	2
Mu and Li (28)	2004	1	1	0	0	2
Meng et al. (29)	2005	2	1	0	0	3
Zhang Guijuan (30)	2006	1	1	0	0	2
Zhang (36)	2006	1	1	0	0	2
Tian et al. (44)	2007	1	1	0	0	2
Ding et al. (17)	2009	2	1	0	1	4
Chen et al. (37)	2010	2	2	0	0	4
Liu and Dong (31)	2010	1	1	0	0	2
Zhu et al. (32)	2010	1	1	0	0	2
Shimeng (26)	2011	2	1	0	1	4
Liu et al. (38)	2013	1	1	0	1	3
Zhai (33)	2013	1	1	0	0	2
Zhang et al. (39)	2015	2	2	2	1	7
Wen (40)	2016	1	1	0	1	3
Li (41)	2017	1	1	0	1	3
Tan et al. (42)	2019	2	1	0	0	3
Lin et al. (43)	2020	2	1	0	1	4
Wang et al. (34)	2020	2	2	2	1	7
Yang (15)	2020	2	1	0	1	4
Chenggang (35)	2022	2	2	0	1	5

### 3.4 Meta-analysis results

#### 3.4.1 Effectiveness

**Figure 2:**
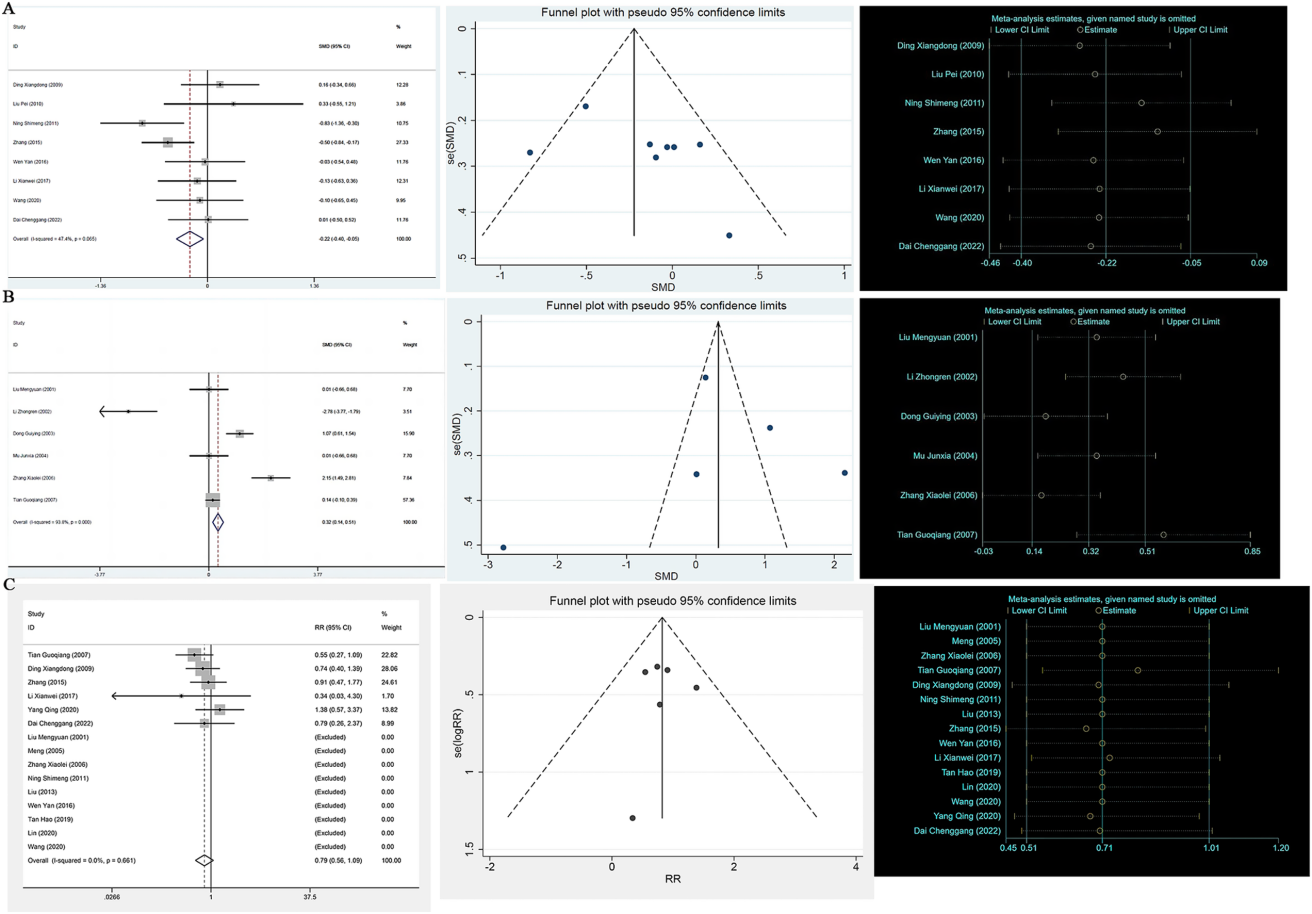
**(A)** Efficient forest map, funnel map, sensitivity analysis; **(B)** MMSE forest map, funnel map, sensitivity analysis; **(C)** ADL forest map, funnel map, sensitivity analysis.

A heterogeneity test analysis showed no statistical heterogeneity among the 16 studies (*p* = 0.085, *I*^2^ = 34.7%); therefore, a fixed effect model was used for combined analysis. The results of the meta-analysis (Figure 2A) show that in terms of overall effective rate, Chinese herbal compounds were superior to Western medicine in the treatment of AD (RR = 1.19, 95% CI: 1.04, 1.37, *p* = 0.009). To test the stability of the results, a sensitivity analysis was performed, and the results did not change significantly. In addition, Egger’s test (*T* = 2.33, *p* = 0.035) and the funnel plot results show that there was no significant publication bias.

#### 3.4.2 Mini-mental state examination

A total of 19 studies reported MMSE scores. A heterogeneity test analysis showed that there was statistical heterogeneity among 19 studies (*p* < 0.001, *I*^2^ = 81.5%); therefore, a random effect model was used for combined analysis. The results of the meta-analysis showed no significant difference in MMSE score between TCM compounds and Western medicine in the treatment of AD (SMD = 0.18, 95% CI: −0.08, 0.44, *p* = 0.180) (see Figure 2B). To test the stability of the results of the study, a sensitivity analysis was performed, and the results did not change significantly. In addition, Egger’s test (*T* = 1.35, *p* = 0.195) and the funnel plot results show that there was no significant publication bias.

#### 3.4.3 Activities of daily living

A total of 11 studies reported ADL scores. A heterogeneity test analysis showed statistical heterogeneity among 11 studies (*p* < 0.001, *I*^2^ = 74.3%); therefore, a random effect model was used for combined analysis. The results of the meta-analysis showed no significant difference in ADL score between TCM compounds and Western medicine in the treatment of AD (SMD = −0.25, 95% CI: −0.52, 0.03, *p* = 0.076) (see Figure 2C). To test the stability of the results of the study, a sensitivity analysis was performed, and the results did not change significantly. In addition, Egger’s test (*T* = −1.19, *p* = 0.263) and the funnel plot results show that there was no significant publication bias (see Figure 2C).

#### 3.4.4 Alzheimer’s disease assessment scale-cognitive

**Figure 3:**
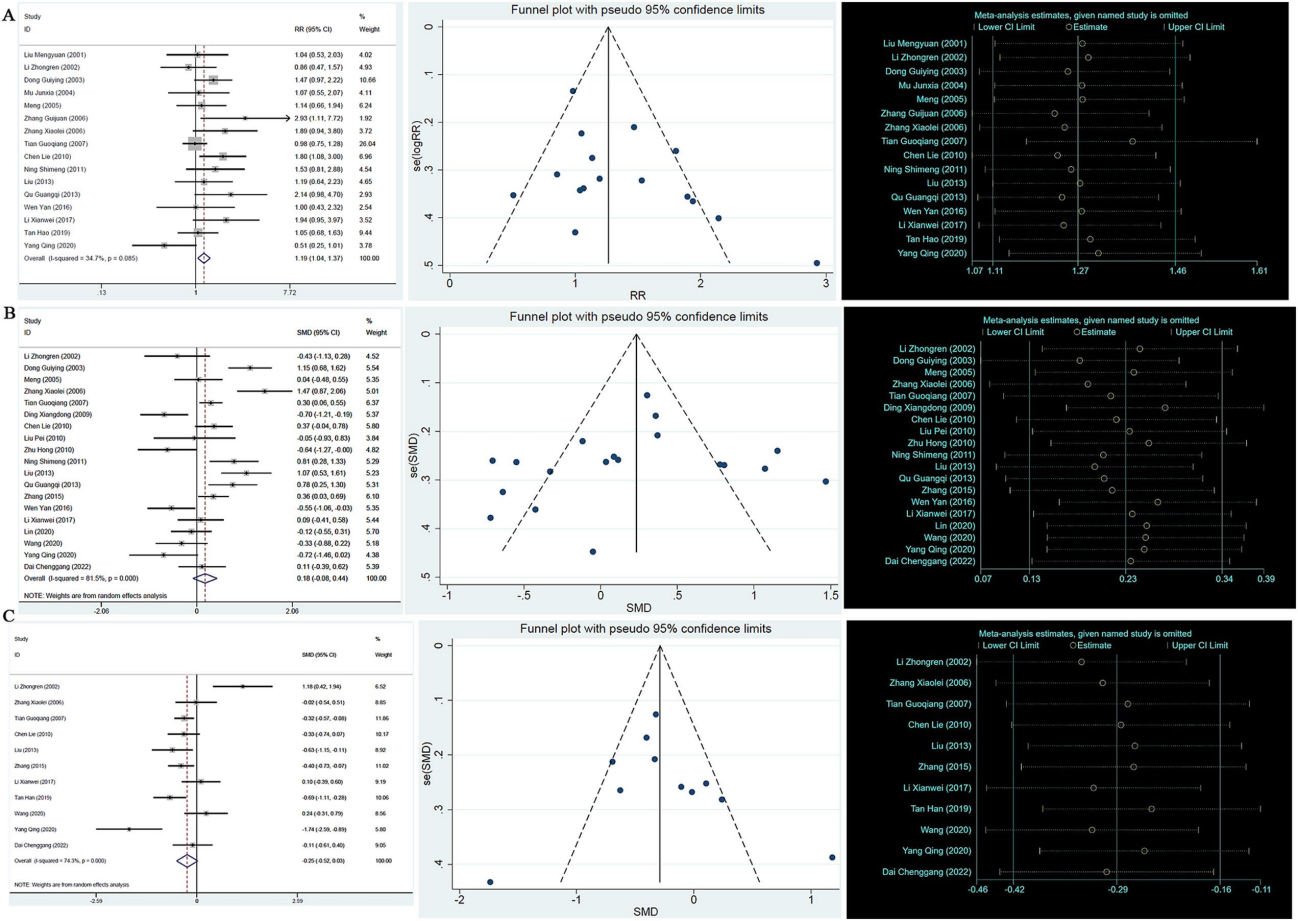
**(A)** ADAS-cog forest map, funnel map, sensitivity analysis; **(B)** HDS-R forest map, funnel map, sensitivity analysis; **(C)** Adverse reaction forest map, funnel map, sensitivity analysis.

A total of eight studies reported the ADAS-cog score. A heterogeneity test analysis showed that no statistical heterogeneity existed between the eight studies (*p* = 0.065, *I*^2^ = 47.4%); therefore, a fixed effect model was used for combined analysis. The results of the meta-analysis showed that the ADAS-cog score of TCM compounds in the treatment of AD was improved compared with Western medicine (SMD = −0.22, 95% CI: −0.40, −0.05, *p* = 0.012) (see Supplementary Table 1). To test the stability of the results of the study, a sensitivity analysis was performed, and the results did not change significantly. In addition, Egger’s test (T = −1.98, *p* = 0.095) and the funnel plot results show that there was no significant publication bias (see Figure 3A).

#### 3.4.5 Hierarchic dementia scale-revised

A heterogeneity test analysis showed that there was statistical heterogeneity among the six studies (*p* < 0.001, *I*^2^ = 93.8%); therefore, a random effect model was used for combined analysis. The results of the meta-analysis showed that the Hierarchic Dementia Scale-Revised (HDS-R) score of TCM compounds in the treatment of AD was improved compared with Western medicine (SMD = 0.32, 95% CI: 0.14 ~ 0.51, *p* < 0.001) (see Supplementary Table 1). To test the stability of the results, a sensitivity analysis was performed, and the results showed no significant change. In addition, Egger’s test (*T* = 0.51, *p* = 0.639) and the funnel plot results show that there was no significant publication bias (see Figure 3B).

#### 3.4.6 Adverse reactions

The occurrence or otherwise of adverse reactions was recorded in 15 studies. In the control group, nine studies recorded no adverse reactions during treatment, and six studies recorded adverse reactions. A heterogeneity test analysis showed no statistical heterogeneity among the six studies (*p* = 0.661, *I*^2^ = 0); therefore, a fixed effect model was used for combined analysis. The results of the meta-analysis (Supplementary Table 2) showed no significant difference in adverse reactions between TCM compounds and Western medicine in the treatment of AD (RR = 0.79, 95% CI: 0.56, 1.09, *p* = 0.155). To test the stability of the results, a sensitivity analysis was performed, and the results did not change significantly. In addition, Egger’s test (*T* = 2.13, *p* = 0.100) and the funnel plot results show that there was no significant publication bias (see Figure 3C).

## 4 Discussion

AD is a common disease and an important cause of death in older adults (45), and it is still incurable. Western medicine treatment improves symptoms, but the long-term effect is not ideal. As the disease worsens, the symptoms are complex and diverse, including multiple mental and behavioral symptoms, which bring challenges to clinical treatment and home care (46). The pathogenesis of AD is not yet fully understood. Traditional Chinese medicine believes that spleen and kidney deficiency, spleen deficiency, and phlegm obstruction are the fundamental syndromes of AD, and kidney deficiency is the primary pathogenesis. Therefore, TCM treatment of AD primarily uses TCM decoction for tonifying the kidney and benefiting intelligence (10, 11). The basic and clinical research of TCM in the treatment of AD has achieved certain results (34, 38, 43). The results show that, compared with donepezil, Bushen Huoxue prescription has improved the daily living ability of patients with AD, and no obvious adverse reactions have been found (47). In addition, some studies have shown that both single Chinese medicine and single effective components of Chinese medicine have played a positive role in promoting research into AD (48). However, the sample size of single clinical trials is often small, the test methods are different, and the quality is uneven. The effect of TCM compounds in the treatment of AD is better than that of Western medicine, and there is little supporting evidence. Therefore, through a systematic review and meta-analysis, this study examined the published randomized controlled trials of TCM compounds and single Western medicine in the treatment of AD and discussed their relative efficacy. The aim was to provide a foundation for the clinical application of TCM compounds, thereby providing a reference for the development of an AD treatment program.

The 23 studies included in this study showed that TCM compounds had a good clinical and maintenance effect in the treatment of AD. The results indicate that the effective rate of TCM compounds in the treatment of AD was higher than that of Western medicine. The ADAS-cog and HDS-R scores of the two groups showed that the curative effect of TCM compounds in the treatment of AD was also better than that of Western medicine. No significant difference was found in ADL and MMSE scores between the two groups, indicating no significant difference in the improvement of cognitive and daily living abilities. In addition, there was no significant difference in adverse reactions between TCM compounds and Western medicine in treating AD, and no obvious adverse reactions or drug side effects were observed.

There are certain limitations in this study. First, the research participants included in the literature are all from China, and the universality of the research results is limited, which may affect the extrapolation of the results. Second, the TCM compound prescriptions are not unified and cannot be carefully distinguished, which has certain limitations for clinical medication guidance. Some of the included studies did not use blinding, which may lead to some bias; a small number of studies were included, and the quality of some studies is low, which may have an impact on the results. To summarize, the present research indicates that TCM compounds could be a promising therapeutic option for AD, demonstrating encouraging results in terms of efficacy and safety, particularly regarding certain cognitive functions. To ensure the reliability of the evidence, it is necessary to conduct more well-designed, unified studies, with fewer confounding factors, a large sample size, and multicenter randomized controlled trials to verify the efficacy of TCM compounds in the treatment of AD.

## Data Availability

The original contributions presented in the study are included in the article/Supplementary material, further inquiries can be directed to the corresponding author.
