# Supplementary material for: Efficacy and safety of traditional Chinese medicine and Western medicine in Alzheimer’s disease: a systematic review and meta-analysis
Source: Front Neurol. 2025 Sep 15;16:1607945. doi: 10.3389/fneur.2025.1607945 (PMC12477022; doi:10.3389/fneur.2025.1607945)
Supplement: Supplementary file 1 [file Table_1.doc]

**Supplement Table 1 Observation of adverse reactions**

| Author | Publishing time | Adverse reaction |
| --- | --- | --- |
| Liu Mengyuan | 2001 | Not mentioned |
| Meng RS | 2005 | For observation of adverse reactions，the three main routine tests (blood，urine and stool)，changes of liver and kidney function，as well asthe subjective complaints by patients such as nau-sea，vomiting，abdominal pain and hallucination,were watched. |
| Zhang Xiaolei | 2006 | Observe whether there are any uncomfortable reactions such as nausea, vomiting, abdominal pain, diarrhea, fall, syncope, blurred vision, headache, etc. after taking the medicine, and observe whether there are any toxic and side effects of the medicine in combination with the above-mentioned examinations and tests. |
| Tian Guoqiang | 2007 | Not mentioned |
| Ning Shimeng | 2011 | General physical examination items: including respiration, heart rate, blood pressure, etc. Blood routine; Urine routine; Routine stool and occult blood; Liver function (ALT, AST) and renal function; Electrocardiogram; Any possible adverse reactions. |
| Liu P | 2013 | Not mentioned |
| Zhang Y | 2015 | The safety parameters including spontaneously reported adverse events (AEs) or serious AEs (SAEs), vital signs (temperature, heart rate and blood pressure), physical examination, and laboratory tests were assessed during each visit. |
| Wen Yan | 2016 | Not mentioned |
| Li Xianwei | 2017 | Including blood routine, urine routine, stool routine, liver function, renal function and electrocardiogram, were tested once before treatment and 12 weeks after treatment, and the possible adverse reactions during medication were observed and recorded in detail. |
| Tan Hao | 2019 | Not mentioned |
| Lin ZY | 2020 | Blood routine，urine routine，stool routine, liver and kidney functions, blood biochemistry and electrocardiogram were detected before and aftertreatment. |
| Wang HC | 2020 | All adverse events，serious adverse events, and concomitant medication use were recorded throughout the study. Vital signs，electrocardiogram, routine laboratory tests，including whole blood count，complete chemistry panel, urinalysis, physical and neurological examinations, were carried out at baseline and at6 months. |
| Yang Qing | 2020 | Test indicators (blood routine, liver and kidney function, blood sugar, blood lipid, electrolyte, stool routine, etc.), routine examination (electrocardiogram, chest X-ray) |
| Dai Chenggang | 2022 | Three routines, liver and kidney function, electrocardiogram and coagulation function |
