# Supplementary material for: Efficacy and safety of traditional Chinese medicine and Western medicine in Alzheimer’s disease: a systematic review and meta-analysis
Source: Front Neurol. 2025 Sep 15;16:1607945. doi: 10.3389/fneur.2025.1607945 (PMC12477022; doi:10.3389/fneur.2025.1607945)
Supplement: Supplementary file 2 [file Table_2.doc]

**Supplement Table 2** Meta-analysis of traditional Chinese medicine compound in the treatment of AD

| Outcome index | N | Heterogeneity | | Pooled Analysis | | | Egger’s Test | |
| --- | --- | --- | --- | --- | --- | --- | --- | --- |
|  |  | *I^2^*(%) | *P*-value | RR/SMD | 95%CI | *P*-vaule | T-vaule | *P*-vaule |
| Effective rate | 16 | 34.7 | 0.085 | 1.19 | 1.04～1.37 | 0.009 | 2.33 | 0.035 |
| MMSE | 19 | 81.5 | <0.001 | 0.18 | -0.08～0.44 | 0.180 | 1.35 | 0.195 |
| ADL | 11 | 74.3 | <0.001 | -0.25 | -0.52～0.03 | 0.076 | -1.19 | 0.263 |
| ADAS | 8 | 47.4 | 0.065 | -0.22 | -0.40～-0.05 | 0.012 | -1.98 | 0.095 |
| HDR | 6 | 93 | <0.001 | 0.32 | 0.14～0.15 | <0.001 | 0.51 | 0.639 |
| Adverse reaction | 6 | 0 | 0.661 | 0.79 | 0.56～1.09 | 0.155 | 2.13 | 0.100 |
